# Non-invasive brain stimulation for the treatment of brain diseases in childhood and adolescence: state of the art, current limits and future challenges

**DOI:** 10.3389/fnsys.2013.00094

**Published:** 2013-11-25

**Authors:** Carmelo M. Vicario, Michael A. Nitsche

**Affiliations:** ^1^School of Psychology, The University of QueenslandSt. Lucia, QLD, Australia; ^2^Clinic for Clinical Neurophysiology, University Medical CenterGöttingen, Germany

**Keywords:** pediatric brain stimulation, vascular diseases, epilepsy, ADHD, Tourette, autism, depression, schizophrenia

## Abstract

In the last decades interest in application of non-invasive brain stimulation for enhancing neural functions is growing continuously. However, the use of such techniques in pediatric populations remains rather limited and mainly confined to the treatment of severe neurological and psychiatric diseases. In this article we provide a complete review of non-invasive brain stimulation studies conducted in pediatric populations. We also provide a brief discussion about the current limitations and future directions in a field of research still very young and full of issues to be explored.

## Introduction

In the last decades the interest in exploring therapeutic and/or rehabilitative effects generated by non-invasive brain stimulation techniques in neuropsychiatric diseases increased considerably. Although this field of research encompasses numerous stimulation techniques, neuroscientists have primarily focused investigations on the use of two techniques, namely Transcranial Magnetic Stimulation (TMS) and transcranial Direct Current Stimulation (tDCS). In healthy adults, these non-invasive brain stimulation techniques are applied to monitor cortical excitability/dynamics (e.g., single pulse TMS, double pulse TMS), and as neuromodulatory techniques [e.g., repetitive TMS (rTMS), tDCS]. Whereas TMS is primarily used to investigate brain physiology (e.g., task-dependent alterations of cortical maps, excitability, and so on, in health, and disease), the latter techniques are applied to *modify* physiology, and performance. Accordingly, these methods have been adopted to explore, and modulate brain functions such as language (e.g., *rTMS*: Flöel et al., 2008; *tDCS*: Vicario and Rumiati, 2012;), learning, and long-term memory formation [e.g., single pulse *TMS* (spTMS): Vicario et al., 2013a; *tDCS*: Nitsche et al., 2003], working memory (WM) (e.g., *TMS*: Gaudeau-Bosma et al., 2013; *tDCS*: Fregni et al., 2005a) perception (e.g., *TMS*: Hamilton et al., 2013; *tDCS*: Vicario et al., 2013b), and attention (e.g., *TMS*: Lee et al., 2013; *tDCS*: Tanoue et al., 2013), in healthy humans (see Nitsche et al., 2003, 2008; Kuo and Nitsche, 2012 for further examples and Nitsche and Paulus, 2011 for a complete review).

Non-invasive brain stimulation is applied also to rehabilitate cortical functions in neuropsychiatric diseases via induction of neuroplasticity. rTMS has been shown to improve cognitive functions in Parkinson's disease (Pascual-Leone et al., 1994), and improved aphasia (Naeser et al., 2005), motor control after stroke (Takeuchi et al., 2005), epilepsy (see Nitsche and Paulus, 2009 for a review), and depression (Pascual-Leone et al., 1996; O'Reardon et al., 2007), amongst others (Kammer and Spitzer, 2012).

Encouraging clinical effects have been documented also for tDCS, so far in pilot studies. tDCS improves motor and non-motor stroke symptoms (e.g., Fregni et al., 2005b), depression, and might have effects on craving, schizophrenia, and dementia, amongst others (for comprehensive reviews, see, Flöel, 2013; Krause et al., 2013; Kuo et al., 2013). All these results encourage the future therapeutic application of non-invasive brain stimulation techniques also in pediatric populations affected by brain disorders. However, the number of non-invasive brain stimulation studies conducted in childhood is scanty, especially if compared to that available from adult participants.

Two previous reviews (Quintana, 2005; Croarkin et al., 2011) gave an overview of the application of rTMS in children and adolescents. Here, we provide the reader with an updated state of the art of application of non-invasive brain stimulation in general (rTMS and tDCS) in pediatric populations. Moreover, we will discuss current limits and possible future applications of these techniques for the treatment of brain dysfunctions affecting childhood. It is beyond the scope of this review to discuss the mechanisms of how TMS and tDCS alter neural activity and induce brain plasticity in detail. For obtaining this information, some recently published articles provide a complete and exhaustive overview (see for example Stagg and Nitsche, 2011; Freitas et al., 2013; Krause et al., 2013).

## Non-invasive brain stimulation for enhancing brain disorders in childhood

### Vascular diseases

Pathophysiologically, stroke is associated with hemispheric dysbalance, i.e., a reduction of the activity of the lesioned brain area and an enhanced activity of the contra-lesional homologous region, which limits re-gain of functions (see Grefkes and Fink, 2011 for a recent review).

Kirton et al. (2008) conducted the first randomized sham-controlled rTMS trial in children (median age 13.25) affected by arterial ischemic stroke. Patients were affected by unilateral hand motor weakness. They received 8 days to 1 Hz rTMS of the contralesional motor cortex, which inhibits regional brain activity (Pascual-Leone et al., 2005) and increases contralateral cortical excitability via reduction of interhemispheric inhibition (Pal et al., 2005). Grip strength increased after real rTMS only, and this effect persisted until day 17 after the start of treatment. Furthermore, rTMS was well tolerated with no relevant side effects.

Valle and colleagues (2007) applied a 5-day course of rTMS upon the affected motor cortex in seventeen children (mean age 9) affected by infantile cerebral palsy and spastic quadriplegia. The study was designed according to the hypothesis that an increase of motor cortex activity would increase its inhibitory influence on spinal excitability, and thus improve spasticity. Patients received five consecutive sessions of rTMS in a randomized, sham controlled, double-blind, parallel, clinical trial design. According to previous works (e.g., Quartarone et al., 2005) showing that 5 Hz rTMS of the primary motor cortex induces an overall increase of excitability of the corticospinal output system, including spinal motoneurons, the authors report a significant reduction of spasticity only in association to this stimulation protocol. Both studies show a potential of rTMS for rehabilitation of motor symptoms originating from vascular injuries in childhood. However, due to the limited number of studies, we are still far from achieving a clear picture of the use of this technique to treat vascular problems in children. No studies for the treatment of vascular disorders in children or adolescents have been conducted with tDCS so far.

### Epilepsy

The pathophysiological substrate of epilepsies and the proneness to develop seizures is an enhanced cortical excitability, leading to paroxysmal depolarization shifts, an enhanced probability of high-frequent and hyper-synchronous activity of neuronal networks (Stafstrom, 2006; Dudek and Sutula, 2007). A reduction of neuronal excitability is the common aim of antiepileptic therapies.

Fregni et al. (2005c), tested the effect of a single 0.5 Hz rTMS session in an open study. Three pediatric patients with focal epilepsy were included. The TMS coil was positioned over the epileptogenic region, or, if this could not be clearly identified, over Cz. rTMS significantly reduced the frequency of epileptiform discharges (ED) for up to 15 and 30 days after rTMS treatment.

The study of Brasil-Neto et al. (2004) included a 6 years old patient. This patient was affected by left fronto-central slow activity. 0.3 Hz rTMS was applied once per day for 3 months. In this case, rTMS treatment was ineffective. Graff-Guerrero et al. (2004) conducted one session of 20 Hz rTMS in two patients (7 and 11 years old) suffering from epilepsia partialis continua (EPC). The patients were first submitted to a single photon emission computed tomography (SPECT) session in order to localize the focal frontal hyperperfused region to define the stimulation site; after rTMS, patients were SPECT-monitored again in order to identify perfusion alterations of the stimulated region. Indeed, cortical perfusion of the stimulated area was reduced in both patients. However, only in one patient epileptic seizures decreased significantly. Morales et al. (2005) conducted a case study involving 2 patients (8 and 16 years old) affected by EPC. One Hertz/six Hertz rTMS were applied in different sessions. No adverse effects occurred, but the treatment resulted in no clinical effects. Kinoshita et al. (2005) treated a 16 years old male suffering from parietal lobe epilepsy with 0.9 Hz rTMS over 5 days. The coil was placed over PCz. No significant improvements have been documented for this patient.

San-Juan et al. (2011) applied cathodal tDCS over the F2 scalp site in a 17 years old patient affected by Rasmussen's encephalitis [4 tDCS sessions of 60 min (on days 0, 7, 30, and 60)]. At follow-up evaluations 6 and 12 months later, seizure frequency was significantly reduced. Additionally, the patient had improved levels of alertness and language. No side effects have been reported. Yook et al. (2011) applied cathodal tDCS applied upon the epileptogenic focus in a 11 year's old patient. The patient was diagnosed with congenital bilateral perisylvian syndrome. Cathodal tDCS was applied over the right temporo-parietal area that showed epileptiform activity in the EEG for 2 weeks. During the two-month period after treatment termination, only six seizures occurred (compared to eight seizures a month before the treatment), and seizure duration decreased. tDCS was repeated for another 2 weeks, 2 months after the first intervention session. For the following two months, only one seizure occurred. No notable side effects of stimulation were observed.

In contrast, in a group of pediatric patients (age range 6–11) showing continuous spikes and slow waves during sleep (CSWS), one session of cathodal tDCS over the peak negativity of the epileptogenic pattern revealed an effect on EEG patterns only in 3 patients (Varga et al., 2011). One reason for this negative result might be that the multifocal/diffuse and poorly defined origin of epileptic activity in CSWS makes it difficult to identify the optimal region for stimulation (Brazzo et al., 2012). Moreover, stimulation was performed with 1 mA in only one session in the latter study, which might have not been sufficient to obtain significant or lasting effects.

More recently, Auvichayapat et al. (2013) enrolled thirty six children (age range 6–15 years) with focal epilepsy in a crossover sham-controlled study. Participants received a single session of cathodal tDCS upon the epileptogenic region. Active tDCS treatment was associated with significant reductions in epileptic discharge frequency immediately and 24 and 48 h after tDCS. Moreover, 4 weeks after treatment, a small (clinically negligible but statistically significant) decrease in seizure frequency was detected. All patients tolerated tDCS well.

These studies deliver some preliminary hints for a possible efficacy of rTMS, and tDCS, to treat epilepsy in children. However, the heterogeneity of the results, possibly due to different stimulation parameters, small numbers, and different etiologies of participants, and the heterogeneous quality of the studies, do not allow to draw definite conclusions.

### ADHD

Attention-deficit/hyperactivity disorder (ADHD) is a highly prevalent and impairing disorder, characterized by inattention, hyperactivity, and executive dysfunction. Functional neuroimaging studies have shown functional and abnormalities in cingulate, frontal and parietal cortical regions, including hypoactivation (Bush, 2011). Thus, non-invasive brain stimulation procedures to improve ADHD symptoms are oriented toward excitatory protocols.

Weaver et al. (2008) performed a randomized, sham-controlled, crossover study involving 9 participants affected by ADHD. The age range of the 4 included adolescents was between 15 and 17 years. 10 Hz rTMS over 2 weeks was applied upon the right DLPFC. Clinical global impression and the ADHD-IV scales improved significantly, but similarly for active and sham rTMS. No serious adverse effects did take place. No studies on childhood ADHD have been performed with tDCS so far.

### Tourette syndrome

Tourette's syndrome (TS) is one of the most common neurobehavioral disorders in childhood (see American Academy of Pediatrics, 2000). The pathology is characterized by the presence of tics, which are rapid, stereotyped movements and vocalizations, virtually involving all body segments (Vicario et al., 2010). The neuro-functional profile of childhood TS is characterized by impairment of neural circuits linking the cerebral cortex to the striatum and other sub-cortical regions (Swain et al., 2007; Bush, 2011).

Of interest for the current discussion is the Supplemental Motor Area (SMA). The pre-motor cortex has been reported to be hyperexcitable in patients with TS (George et al., 2001). Therefore, excitability-reducing rTMS to the SMA may be an effective way to treat TS, because this region is extensively connected with regions implicated in motor control (Picard and Strick, 2001).

In an open label 12 weeks cohort pilot study (Kwon et al., 2011), 1Hz rTMS was applied over the SMA for 10 days. At the end of each day subjects completed objective ratings of ADHD, mood, anxiety, tics, and side effects. Statistically significant reductions were seen in the Yale Global TS Severity Scale over the 12 weeks of the study. Le et al. (2013) tested the effect of 1 Hz rTMS upon the SMA in 25 children with TS (aged less than 16 years) for 20 days. Results document a significant improvement of clinical symptoms. Interestingly, the benefits lasted for up to 6 months in 68% of subjects. No studies for TS treatment have been conducted with tDCS. The results provided in these two studies are coherent in terms of therapeutic benefits, stimulation site (SMA) and rTMS frequency (1 Hz). However, an important limit of these studies is the lack of a control group.

### Autism spectrum disorder (ASD)

This syndrome is characterized by a marked decrease in social integration and communication, and affects ~1 in 150 children (Fombonne, 2009). Increased gamma-band responses to several cognitive processes in children with autism spectrum disorder have been described (McFadden et al., 2012). While its precise role is unclear, it is implied in a wide range of processes such as attention (Lakatos et al., 2008), WM (Tallon-Baudry et al., 1998), and language (Tavabi et al., 2011). Non-invasive brain stimulation has been adopted for modulating this physiological parameter and therefore improve the related cognitive abilities.

Baruth et al. (2010) report clinical improvements in pediatric ASD induced via rTMS (≤1 Hz) in a controlled study, the electrophysiological effects of 12 low frequency rTMS sessions, bilaterally applied to the DLPFC were explored in 25 subjects (ages range 9–26) with ASD and 20 age-matched controls. rTMS was administered once per week. The first six treatments were performed over the left, and the remaining six over the right DLPFC. Patients showed significant improvement in discriminatory gamma activity and also significant improvements in behavioral parameters. In another study (Sokhadze et al., 2010) 20 subjects (age range 10–19) with ASD received the same protocol. Performance was tested with the oddball paradigm, which explores attentional shifting (García-Larrea et al., 1992). rTMS minimized early cortical responses to irrelevant stimuli, increased responses to relevant stimuli, reduced the error percentage and repetitive-ritualistic behavior.

Schneider and Hopp (2011) conducted an open tDCS study in children with autism. The purpose was to improve language acquisition in patients with minimal verbal language. In this study the authors selected 10 ASD participants (age range 16–21). Post-anodal tDCS of the Broca area, mean vocabulary scores were significantly higher than the pre-anodal tDCS scores.

The studies are suggestive for therapeutic benefits of non-invasive brain stimulation in autistic children. However, due to the small numbers of studies, in which different protocols had effects on different target functions, statements about optimal protocols are premature.

### Depression

Depression in children and adolescents is associated with significant functional disability in multiple environmental realms (Cosgrove et al., 2013). Depression involves a distributed network of cortical and limbic regions, including the DLPFC (especially the left), and subgenual cingulate gyrus, amongst others (Mayberg, 2007). It has been shown (Fitzgerald et al., 2006) that in depression these areas, and specifically the left-hemispherical ones, may be hypoactive, whereas right-hemispheric hyper-activation might take place, thus constituting an hemispheric dysbalance of activation. The rationale of the brain stimulation studies presented here is to increase the activity of the left DLPFC.

Walter et al. (2001) were the first to report the impact of rTMS on depression in 3 patients younger than 18 years. The patients received daily treatment over 2 weeks of 10 Hz rTMS over the left DLPFC. Two participants improved clinically, but one of them complained about tension headache during two of the treatment sessions. In another case report series, Loo et al. (2006) tested the effect of 10 Hz rTMS upon the left DLPFC over 6 weeks on two 16-year-old adolescent girls affected by depression and ADHD. No improvements of ADHD symptoms, but a reduction of depression symptoms was reported. Bloch et al. (2008) conducted an open label rTMS study with nine adolescents (16–18 age range) affected by severe treatment-resistant depression. Participants received 14 sessions of 10 Hz rTMS to the left DLPFC. Depression scores of the participants improved significantly. Two of them remained in clinical remission 1 year after therapy (as judged by regular clinical follow-up). Five subjects reported mild headaches. No other adverse effects were reported. More recently, Wall et al. (2011) conducted an open-label trial of adjunctive rTMS in eight adolescents with treatment-resistant depression. These subjects were maintained on a stable dose of a selective serotonin reuptake inhibitor (SSRI). All in all, thirty daily 10 Hz rTMS treatments were given over the left dorsolateral prefrontal cortex. The CDRS-Revised mean scores improved significantly from baseline over the course of 30 treatments and the 6-month follow-up. Pre- and post-treatment neurocognitive testing did not reveal any decline in functioning. These data are preliminary as no control group was included, but this study shows that intensive treatment parameters were well tolerated by adolescent patients. No studies for treatment of depression in children have been conducted with tDCS.

The available studies suggest some therapeutic effect of rTMS for the treatment of childhood depression. A recurring element associated with a successful rTMS therapy is the stimulation of left DLPFC at 10Hz. However, since these are open and/or case report studies, the results of these studies should be interpreted with caution.

### Schizophrenia

Childhood-onset schizophrenia is a rare and severe form of the disorder (Nicolson and Rapoport, 1999), that is neurobiologically and physiologically continuous with adult onset schizophrenia (David et al., 2011). Hallucinations are, probably, the most dramatic clinical symptom that causes significant problems for the life of patients with schizophrenia.

Physiological abnormalities have been reported in several neural regions including right medial temporal, lateral temporal, inferior frontal cortex, and in the cingulate cortex bilaterally, left DLPFC and left superior temporal gyrus (Vyas and Gogtay, 2012; Hayempour et al., 2013). However, neuroimaging studies have shown relatively less predictive value despite consistent reports of progressive structural brain abnormalities associated with schizophrenia. An increase of left temporoparietal cortex excitability is associated with positive symptoms, specifically auditory hallucinations (AHs) (Silbersweig et al., 1995; Shergill et al., 2000). On the other hand, activity enhancement of the left prefrontal region has been suggested to improve negative symptoms (Heimer et al., 1997), due to its effect on the release of dopamine (Strafella et al., 2001). Therefore, excitability-reducing stimulation is proposed to reduce activity of the left temporoparietal cortex, while excitatory non-invasive brain stimulation has been used to increase left prefrontal region activation for therapeutic application (see Freitas et al., 2009; Demirtas-Tatlidede et al., 2013 for a complete review).

Jardri et al. (2007) report a single case study involving a 11-year-old boy with medication-resistant schizophrenia. An fMRI scan displayed increased auditory cortex activity with concurrent AHs. 10 sessions of 1 Hz rTMS were administered to the left temporoparietal cortex. Verbal AHs decreased by 50%. The improvement obtained with rTMS was maintained by repeating the sessions every 5 weeks. No adverse effects of rTMS were reported. More recently, the same group (Jardri et al., 2012) established a case series of adolescents diagnosed with childhood-onset schizophrenia according to the Schedule for Affective Disorders and Schizophrenia for School-Age Children (*n* = 10, 15.5 years old). All participants had frequent and drug-resistant AHs. The patients received 1 Hz rTMS to the T3-P3 site over 5 days. The authors assessed scalp discomfort clinically and describe only minor discomfort. AHRS scores decreased significantly from baseline to the immediate post-treatment assessment, and from baseline to the 1-month assessment. Furthermore, the Global Assessment of Functioning scores improved significantly immediately and 1 month after the treatment, as compared to baseline values.

For tDCS, one study was recently published with regard to childhood-onset schizophrenia (Mattai et al., 2011). This study aimed to investigate the tolerability of tDCS in this patient group. Twelve participants (12 children, age range 10–17) were assigned to one of two groups: bilateral anodal DLPFC stimulation (*n* = 8) or bilateral cathodal superior temporal gyrus (STG) stimulation (*n* = 5). The stimulation protocol consisted of 20 min per day, per 2 weeks. No subjects reported significant discomfort at the electrode sites. However, four individuals had transient redness of the skin under the electrodes that resolved within about an hour after treatment. Although no significant clinical improvement has been reported, this study is the first to demonstrate that tDCS with the applied parameters is well tolerated in adolescents. Complains of fatigue reported by some patients could be related to unspecific effects, such as medication regimens that frequently include the atypical antipsychotic clozapine.

Taken together, knowledge about the effects of non-invasive brain stimulation in childhood onset schizophrenia is still limited. However, the rTMS case reports provided by Jardri et al. hint for some efficacy in reducing hallucinations. The only available tDCS study is in favor for good tolerability of this stimulation protocol. Systematic studies are needed to explore this field to a larger degree.

## Discussion

In this work we reviewed the available literature about the effects of non-invasive brain stimulation in pediatric populations (i.e., younger than 18 years) suffering from neuro-psychiatric diseases. We focused our analysis on studies which have examined the therapeutic efficacy of rTMS and tDCS for rehabilitating neurological functions and ameliorating psychiatric syndromes in children.

In general, the studies provide preliminary evidence in support for a therapeutic potential of non-invasive stimulation techniques in children and adolescents. However, several limitations should be taken into account.

Virtually no double-blinded sham controlled studies are available at present, which makes it difficult to make safe statements about efficacy. Since there seems to be a potential, these studies are urgently needed. Moreover, systematic studies to identify optimal stimulation protocols based on physiology are missing, including comparisons between different protocols, and a systematic evaluation of safety of non-invasive brain stimulation with regard to the developing brain. It is important to note that, although the examined literature does deliver no hint for major adverse effects in children, the evidence currently available is still rather scanty. Studies conducted in adult humans and animal models are probably not transferable one-to-one to children, since anatomy and physiology differs relevantly between these groups (Johnston, 2009). In this regard, it is important to consider that the developing brain is characterized by “sensitive” periods or rather periods during which the effects of the experience on the brain are unusually strong during a limited period in development (Knudsen, 2004). This suggests that, during development, the risk to induce maladaptive neural plasticity due to brain stimulation techniques might be relatively high (Vicario and Nitsche, 2013). Because of this, we suggest that neuroscientists willing to apply non-invasive brain stimulation in children should consider dose-finding studies, and a longitudinal monitoring of neural plasticity triggered by these methods (through neuroimaging and/or electrophysiological techniques), to control for functional and structural changes. In this connection, it will be important to improve knowledge about physiological effects of stimulation in children. This might provide important landmarks for assessing the therapeutic effectiveness of the adopted stimulation protocol as well as the presence of side effects. Moreover, discoveries provided by research in “sensitive” periods at the circuit level might be helpful for this issue as they could supply insights of paramount importance for predicting the trajectories of neural plasticity induced by non-invasive brain stimulation techniques and their related therapeutic results. Worthy of discussion is also the fact that the current state of investigations involving the application of brain stimulation methods in pediatric populations is unbalanced toward TMS (See Tables 1, 2 for detailed summaries).

**Table 1:** **rTMS studies in childhood brain diseases**.

**Studies**	**Design**		**Patients**		**Stimulation protocol**						**Outcome**	
	**Sham controlled**	**Blinding**	**Disease**	**Sample size**	**rTMS intensity**	**Stimulation position**	**Number of sessions/days**	**rTMS frequency**	**Total number of pulses/session**	**Duration per session**	**Effects**	**Side-effects**
**VASCULAR DISEASES**												
Kirton et al., 2008	Yes	Partially	AIS	10	100% rMT	Controlesional motor cortex	1 session per day/8 days	1 Hz	1200	20 min	Grip strength improvement	None
Valle et al., 2007	Yes	Double blind	CP	17	90% rMT	Motor cortex	1 session per day/5 days	5 Hz	1500	N/A	Reduction of spasticity	None
**EPILEPSY**												
Brasil-Neto et al., 2004	No	Open	Left front-central slow activity	1	95% rMT	Frontal cortex (Epileptogenic focus)	1 session per day/2 sessions per week/3 months	0.3 Hz	100	N/A	No effects	None
Graff-Guerrero et al., 2004	No	Open	EPC	2	50% of the TMS device power (case 1) 128% rMT (case 2)	Frontal cortex (Epileptogenic focus)	1session/1 day	20 Hz	N/A	N/A	Seizure discharge reduction	None
Fregni et al., 2005a,b,c	No	Open	Cortical malformation	3	65% of the TMS device power	Epileptogenic focus vs. Cz	1 session/1 day	0.5 Hz	600	20 min	Seizure discharge reduction	None
Morales et al., 2005	No	Open	EPC	2	N/A	Motor cortex (Epileptogenic focus)	2 sessions/1 day (case 1) 2 session/1 day + 1 session (second day- case 2)	Patient 1: 1–6 Hz Patient 2: 1: 1 Hz-6 Hz-1 Hz	N/A	N/A	No effects	None
Kinoshita et al., 2005	No	Pilot	Extratemporal lobe epilepsy	1	90% rMT	PCz	2 sessions per day/5 days in a week	0.9 Hz	N/A	15 min	No effects	None
**ADHD**												
Weaver et al., 2008	Yes	Open	ADHD only	4	100% rMT	Right DLPFC	1 session per day/5 days per week/2 weeks	10 Hz	2000 stimuli	N/A	Improved attention in the Sham + active rTMS condition	None
**TOURETTE**												
Kwon et al., 2011	No	Open	TS only	2	100% rMT	SMA	10 daily sessions/10 days	1 Hz	1200	N/A	Tics reduction	None
Le et al., 2013	No	Open	TS only	25	100% rMT	SMA	1 session per day/5 days per week/4 weeks	1 Hz	1200	20 min	Tics reduction	None
**AUTISM SPECTRUM DISORDERS**												
Baruth et al., 2010	Control group	Open	Autistic	22	90% rMT	Bilateral DLPFC	1 session per day/1 day per week/12 weeks	•1 Hz	150	N/A	Reduction of irritability and repetitive behavior	None
Sokhadze et al., 2010	No	Open	Autistic	13	90% rMT	Bilateral DLPF cortices	1 session per day/2 day per week/3 weeks	0.5 Hz	150	N/A	Reduced errors % in the oddball task and ritualistic behaviors	None
**DEPRESSION**												
Walter et al., 2001	No	Open	Unipolar major depression	3	Between 90 and 110% of the rMT	Left DLPFC	1 session per day/5 days per week/2 weeks	10 Hz	1600	N/A	Improvement in 2 cases on 3	Tension headache reported in one case with improvement
Loo et al., 2006	No	Open	Depression/ADHD comorbidity	2	110% rMT	Left DLPF cortex	1 session per day/5 days per week/6 weeks	10 Hz	40 trains/5 s per train	N/A	Improvement measured with the CDRS	None
Wall et al., 2011	No	Open	Depression	8	120% MT	Left DLPF cortex	1 session per day/5 days per week over 6–8 weeks	10 Hz	3000	N/A	Improvement measured with the CDRS	None
Bloch et al., 2008	No	Open	Depression	9	80% MT	Left DLPF cortex	1 session per day over 14 days	10 Hz	20 sessions/2 s per trains	20 min	Improvement measured with the CDRS and BDI	None
**SCHIZOPHRENIA**												
Jardri et al., 2007	No	Open	Schizophrenia	1	100% rMT	left temporoparietal cortex	1 time per day/10 days	1 Hz	1000 stimuli	N/A	The Verbal AHs decreased	None
Jardri et al., 2012	No	Open	Schizophrenia	10	90% rMT	T3-P3	2 times per day/5 days	1 Hz	1200 stimuli	N/A	Improvement measured with the CDRS	Minor discomfort

*Shown are studies dedicated to the treatment of Vascular diseases, Epilepsy, ADHD, Tourette, Autism Spectrum Disorder, Depression, Schizophrenia in childhood populations. Study characteristics, details of the stimulation protocols as well as effects of stimulation, including side effects, are shown. AIS, Arterial ischemic stroke; CP, Cerebral Palsy; EPC, Epilepsia partialis continua; ADHD, Attention-deficit/hyperactivity disorder. Stimulation targets areas are described according to the international 10-20 system*.

**Table 2:** **tDCS for the treatment of childhood brain disorders**.

**Studies**	**Design**		**Patients**		**Stimulation protocol**						**Outcome**	
	**Sham controlled**	**Blinding**		**Polarity**	**Therapeutic electrode position**	**Return electrode position**	**Current strength (mA)**	**Electrode size (cm^2^)**	**Duration (min)/sessions**	**Sample**	**Effects**	**Side-effects**
**EPILEPSY**												
San-Juan et al., 2011	No	No	Rasmussen's encephalitis	C	F2	F8	2	12 mm in length × 0.4 mm diameter	60 min/4 sessions (on days 0, 7, 30, and 60)	1	Cathodal tDCS improves epilepsy, linguistic, and motor functions	None
Yook et al. (2011)	No	No	Slow waves/right hemisphere and intermittent spikes/temporoparietal area	C	Right temporo-parietal area (between P4-T4)	Left orbit	2	25	20 min/5 days per week/2 weeks	1	Cathodal tDCS reduces seizure attacks and duration	None
Varga et al., 2011	Yes	Partially (patients)	(*n* = 1) SP, GTP (*n* = 1) CP (*n* = 1) SP, CP, GTP (*n* = 1) Atonic (*n* = 1) CP, GTC	C/S	*N* = 1 T7 *N* = 2 FT7 *N* = 3 T7 *N* = 4 TP8 *N* = 5 T7 On the area of peak negativity	On the area of peak positivity (more widespread)	1	25	20 min/2 sessions/1 day	5	Effect on EEG patterns or clinical symptoms only in 3 patients	None
Auvichayapat et al., 2013	Yes	Partially (statician)	(*n* = 18) Idiopathic (*n* = 4) MTS (*n* = 2) FCD (*n* = 3) Others	C/S	Seizure focus	Controlateral shoulder	1	35	20 min (one session)	36	Significant reductions in epileptic discharge frequency immediately and 24 and 48 h after tDCS	None
**AUTISM**												
Schneider and Hopp, 2011	No	Partially (statician)	Autism	A	Left DLPFC	Right supraorbital	0.08	25	30 min (one session)	10	Anodal tDCS over left DLPF cortex improved vocabulary score	None
**Schizophrenia**												
Mattai et al., 2011	Yes	Possibility of open treatment	Schizophrenia	C/A/S	Bilateral anodal DLPFC stimulation (*N* = 8) Bilateral cathodal superior temporal gyrus (STG) stimulation (*n* = 5)	Non-dominant forearm	2	25	20 min/5 days per week/2 weeks	12	None	Tingling (*n* = 7); Itching (*n* = 6)

*Shown are studies dedicated to treatment of Vascular diseases, Epilepsy, ADHD, Tourette, Autism Spectrum Disorder, Depression, Schizophrenia in childhood populations. Study characteristics, details of the stimulation protocols as well as effects of stimulation, including side effects, are shown. A, anodal tDCS; C, cathodal tDCS; S, sham tDCS. Stimulation targets areas are described according to the international 10–20 system. Legend: SP, simple partial seizures; GTP, generalized tonic-clonic seizures; CP, complex partial seizures; MTS, Mesial temporal sclerosis; FCD, Focal cortical dysplasia*.

No published studies are currently available with regard to the therapeutic efficacy of tDCS in childhood disorders such as stroke and/or brain injury, TS, ADHD, and Depression. Nevertheless, the interest in applying tDCS for treating pediatric populations is growing.

## Future direction of research

The application of non-invasive brain stimulation for the rehabilitation and/or treatment of children might have the potential advantage of promoting improvements superior to those achievable in adulthood, according to the fact that the developing brain is characterized by “sensitive periods” during which the effects of the experience on the brain are unusually strong for a limited period (Knudsen, 2004). Therefore, the improvement of symptoms via non-invasive stimulation treatment in pediatric age might be more stable, and consistent. However, as discussed above, this same “sensitivity” might also be risky in terms of induction of maladaptive neural plasticity.

In order to minimize this risk, future lines of research should address the following, still limited, field of investigations:

– Exploration, also by using animal models, of the physiological effects of non-invasive brain stimulation in children;
– Systematic exploration of childhood clinical populations to clarify the pathophysiology of the respective diseases, which could be objective to non-invasive brain stimulation. This is important for defining optimal protocols.
– Systematic conduction of “dose-finding,” sham-controlled, double-blinded studies, which will provide important information not available from the open studies.

These aspects will help to clarify, at several levels of complexity, the potential therapeutic efficacy of non-invasive brain stimulation in children, delivering a realistic basis for clinical application of such stimulation protocols on a large scale.

### Conflict of interest statement

The authors declare that the research was conducted in the absence of any commercial or financial relationships that could be construed as a potential conflict of interest.
